# Differences among the Anthocyanin Accumulation Patterns and Related Gene Expression Levels in Red Pears

**DOI:** 10.3390/plants8040100

**Published:** 2019-04-16

**Authors:** Meng Wu, Jianlong Liu, Linyan Song, Xieyu Li, Liu Cong, Rongrong Yue, Chengquan Yang, Zhuo Liu, Lingfei Xu, Zhigang Wang

**Affiliations:** College of Horticulture, Northwest A&F University, Taicheng Road NO. 3, Yangling 712100, Shanxi Province, China; wumeng530@nwafu.edu.cn (M.W.); pearliu@nwafu.edu.cn (J.L.); linyans@yeah.net (L.S.); lixieyu@nwafu.edu.com (X.L.); imcongliu@163.com (L.C.); yuerongrong@nwafu.edu.cn (R.Y.); cqyang@nwsuaf.edu.cn (C.Y.); 15591861881@163.com (Z.L.)

**Keywords:** red pear, anthocyanin, gene expression, coloration

## Abstract

Differences in coloration exist among red pear cultivars. Here, we selected six red pear cultivars with different genetic backgrounds to elucidate the characteristics of fruit pigmentation. We detected anthocyanin contents and the expression levels of anthocyanin synthesis-related genes in these cultivars at different stages of fruit development. The anthocyanin contents of all six cultivars showed a rise–drop tendency. Principal component and hierarchical cluster analyses were used to distinguish the types of cultivars and the genes crucial to each anthocyanin accumulation pattern. The six cultivars were divided into three groups. Red Zaosu were clustered into one group, Red Sichou and Starkrimson into another group, and Palacer, Red Bartlett, and 5 Hao clustered into a third group. The expression levels of *F3H*, *UFGT2*, *MYB10*, and *bHLH3* were similar among the differential coloration patterns of the six cultivars, suggesting a critical and coordinated mechanism for anthocyanin synthesis. Anthocyanin transporters (*GST*) and light-responsive genes, such as *COP1*, *PIF3.1,* and *PIF3.2* played limited roles in the regulation of anthocyanin accumulation. This study provides novel insights into the regulation of anthocyanins synthesis and accumulation in red pears.

## 1. Introduction

Pears are an important fruit worldwide. The color of the pear is an important factor in consumer acceptance of fresh pears. However, pears show different coloring patterns owing to different genetic backgrounds. At present, there are four major pear classifications in the world, including the Asian pear species (*Pyrus pyrifolia*, *Pyrus bretschneideri*, and *Pyrus ussuriensis*) and European pear species (*Pyrus communis*). Red pears are mostly classified as European pears, while the coloration of most Asian pear cultivars ranges from yellow-green to brown, with a few being red [1]. Little is known about the coloring mechanisms of different red pear cultivars. Thus, it is important to study the mechanisms responsible for the different coloring patterns of red pears. 

The main components of the red color in pears are anthocyanins [2,3,4]. The well-characterized anthocyanin biosynthetic pathway is a highly conserved network found in many plant species [5,6,7,8,9] (Figure S1). Anthocyanin accumulation is controlled coordinately by structural and regulatory genes. Additionally, the structural genes are regulated by the *MYB-bHLH-WD40* complex, consisting of *MYB*, basic helix-loop-helix (*bHLH*) and *WD40* repeat families [10,11,12,13]. In addition, anthocyanin synthesis is controlled by external environmental factors, such as nutrient depletion, drought, pathogen infection, temperature, and light [14]. Light and temperature are decisive environmental factor that affect the accumulation of anthocyanins in fruit. Generally, light can increase the anthocyanin accumulation [15]. Higher temperatures lead to a decrease in anthocyanin accumulation, while low temperatures promote anthocyanin accumulation [16]. Anthocyanin synthesis and degradation occur simultaneously. It is generally believed that anthocyanin degradation occurs as a result of plant-specific developmental stages or changes in environmental factors [17]. Planta degradation of anthocyanins may occur as a result of active enzyme-driven breakdown processes. The color of the *Brunfelsia calycina* flower changed rapidly from deep purple to completely white after flowering, a vacuolar class III peroxidase (BcPrx01) was suggested to be responsible for anthocyanin degradation [18,19]. Some light-responsive genes play important roles in anthocyanin accumulation. In apples (*Malus* × *domestica*), MdCOP1 degrades the MdMYB1 protein, resulting in the inhibition of fruit coloration in a light-dependent manner [20]. Phytochrome-interacting factor 3 (*PIF3*) is a bHLH transcription factor, having critical roles in light signaling [21]. *PIF3* and *HY5* promote anthocyanin accumulation by simultaneously binding the promoters of structural genes at separate sequence elements in Arabidopsis [22]. The anthocyanin is synthesized in the cytoplasm and finally stored in the vacuole, so anthocyanin transport can affect the accumulation of anthocyanin content. GST is an anthocyanins synthesis-related transporter. It was first discovered in maize, in which it was involved in the transfer of anthocyanins from the endoplasmic reticulum to the vacuole [23]. GST has a positive correlation with anthocyanin accumulations in different species [24,25,26,27].

In pear species, the structural genes for anthocyanin biosynthesis, such as *PAL*, *CHS*, *CHI*, *F3H*, *DFR*, *ANS*, and *UFGT* have been isolated [28,29]. *ANS* and *UFGT* are crucial genes for anthocyanin synthesis in Asian and European pears [30]. *PAL* is not a key gene in anthocyanin biosynthesis because anthocyanin accumulation does not change with *PAL* activity [31]. Some transcription factors are also involved in the regulation of anthocyanin biosynthesis in pears. *P. pyrifolia MYB10 (PyMYB10)* plays a role in regulating anthocyanin biosynthesis, and *P. communis MYB10* (*PcMYB10*) could regulate the accumulation of anthocyanins by associating with the promoter of *UFGT* [32,33]. Furthermore, *P. bretschneideri MYB10* (*PbMYB10b*) regulates anthocyanins by inducing the expression of *PbDFR*, and *PbMYB9* induces the synthesis of anthocyanins by binding the *PbUFGT1* promoter in pear fruit [34]. In addition, the expression levels of *MYB10* and *bHLH33* were significantly correlated in European pears but not in Asian pears [30].

The regulatory pathway of anthocyanin synthesis is very complicated. The anthocyanin synthesis pathway has been widely studied in pears. At present, there is limited research on anthocyanin accumulation patterns in different red pears. To study the different coloring patterns of the red pear cultivars, we investigated the different stages of fruit development in six cultivars, 5 Hao, ‘Red Zaosu’, Red Sichou, Palacer, Starkrimson and Red Bartlett. 5 Hao and Red Zaosu are Asian pear species, Red Sichou, Palacer, Starkrimson and Red Bartlett are European pear species. Different red pear cultivars were classified according to the difference in gene expression levels. We aimed to identify key genes that control coloration during fruit development in red pears. The study provides comprehensive insights into the molecular mechanism of anthocyanin accumulation in red pears, especially in the different species of red pear at different stages of fruit development, and provides novel insights into the regulation of anthocyanins.

## 2. Results

### 2.1. Total Anthocyanin Accumulation in Different Pear Cultivars

**Figure 1:**
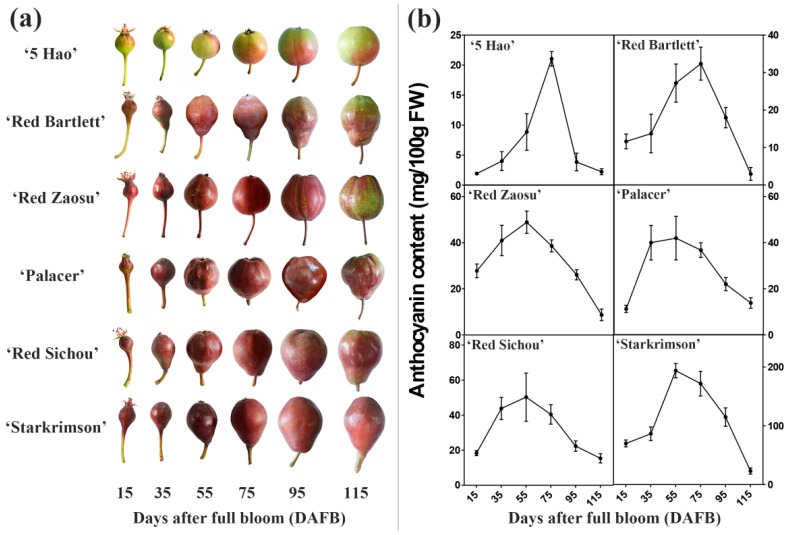
Changes in six red pear cultivars’ skin color and anthocyanin contents at different stages of fruit development. (**a**) The phenotypes and changes in skin color at different stages of fruit development. (**b**) The total anthocyanin contents at different stages of fruit development. Data are means ± standard deviations of three biological replicates.

5 Hao is a blushed red pear, Starkrimson, Palacer and Red Sichou are fully red pears, and Red Bartlett has full-red fruit in the early stages of fruit development that subsequently fade to red-green after maturity [35]. Red Zaosu is a fully red pear at the early stages of fruit development, while its red-green stripes appear at the middle and late stages of fruit development (Figure 1a). The anthocyanin contents of all six cultivars showed a rise–drop tendency (Figure 1b). The anthocyanin content increased gradually in the early stages of fruit development (15 and 35 DAFB), with the highest contents occurring in the middle stages of fruit development (55 and 75 DAFB). In the late stages of fruit development (95 and 115 DAFB), the anthocyanin content began to decline. The anthocyanin contents of 5 Hao and Red Bartlett peaked at 75 DAFB and then declined. In the other four cultivars, the anthocyanin content reached a maximum at 55 DAFB. Among these cultivars, the highest content of anthocyanins in all stages was found in Starkrimson, followed by Red Zaosu, Red Sichou and Palacer, which all had similar anthocyanin contents.

### 2.2. Expression Levels of Anthocyanin Synthesis-Related Structural Genes

**Figure 2:**
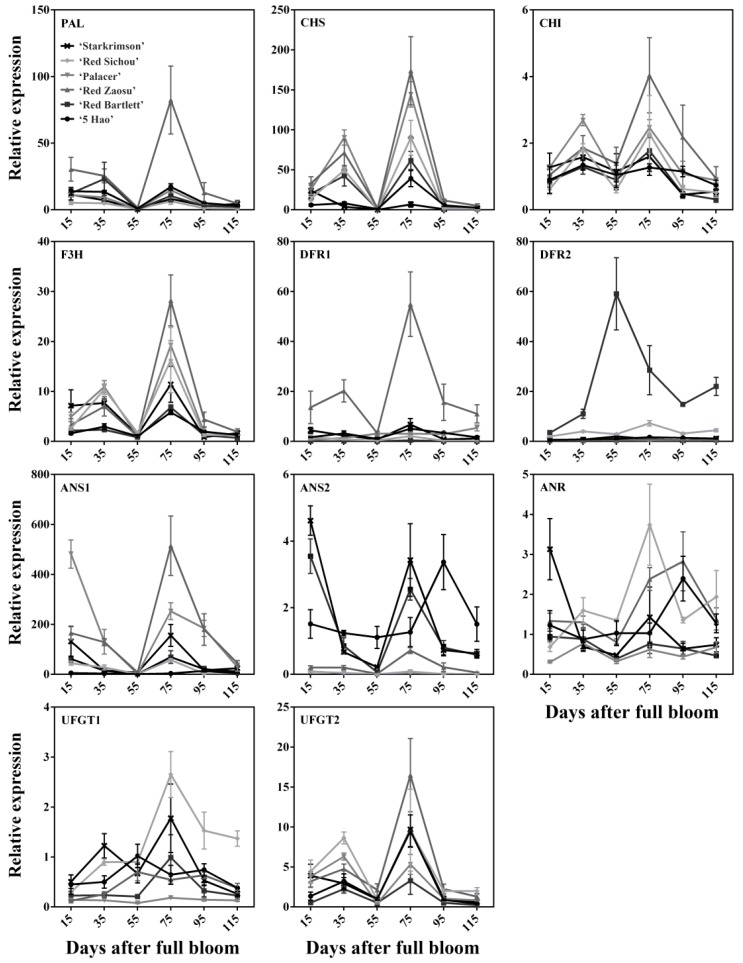
Relative expression levels of the anthocyanin biosynthesis-related structural genes of six red pear cultivars at different stages of fruit development.

To determine the critical genes involved in the regulation of anthocyanin synthesis, the expression levels of structural genes *PAL*, *CHS*, *CHI*, *F3H*, *DFR1*, *DFR2*, *ANS1*, *ANS2*, *ANR*, *UFGT1*, and *UFGT2* were determined by quantitative real-time PCR (qPCR) during the developmental stages (Figure 2). *PAL* showed a peak of expression at 75 DAFB in Red Zaosu. However, *PAL* was highly expressed at the early stages of fruit development in the other cultivars. *CHS*, *F3H*, and *UFGT2* had the same trend, showing their highest expression levels in the six cultivars at 75 DAFB. The trend was basically consistent with the accumulation of anthocyanins. However, the expression level of *CHS* in Starkrimson was an exception—its highest expression levels occurred at 15 DAFB. The relative expression level of *CHI* in all of the developmental stages of the fruit did not change significantly, but was slightly higher at 35 and 75 DAFB than at the other periods. The expression level of *DFR1* in Red Zaosu was much greater than in the other cultivars, while the expression level of *DFR2* was much greater in Red Bartlett than in the other cultivars. The expression levels of *ANS1* and *ANS2* peaked at 75 DAFB in Red Zaosu and peaked at 95 DAFB in 5 Hao. However, in other cultivars, the expression levels of *ANS1* and *ANS2* were higher at 15 and 75 DAFB, and showed a drop–rise–drop tendency that was not consistent with the trend in anthocyanin accumulation. The expression levels of *ANR* did not change much during any of the stages of fruit development. *UFGT1* generally showed the same trend in Red Bartlett, Red Sichou and Starkrimson, with the trend similar to the change in the anthocyanin content. These data indicated that the expression of the *F3H* and *UFGT2* genes have essential roles in the anthocyanin accumulation of the six cultivars.

### 2.3. Expression Levels of Anthocyanin Synthesis-Related Regulatory Genes 

**Figure 3:**
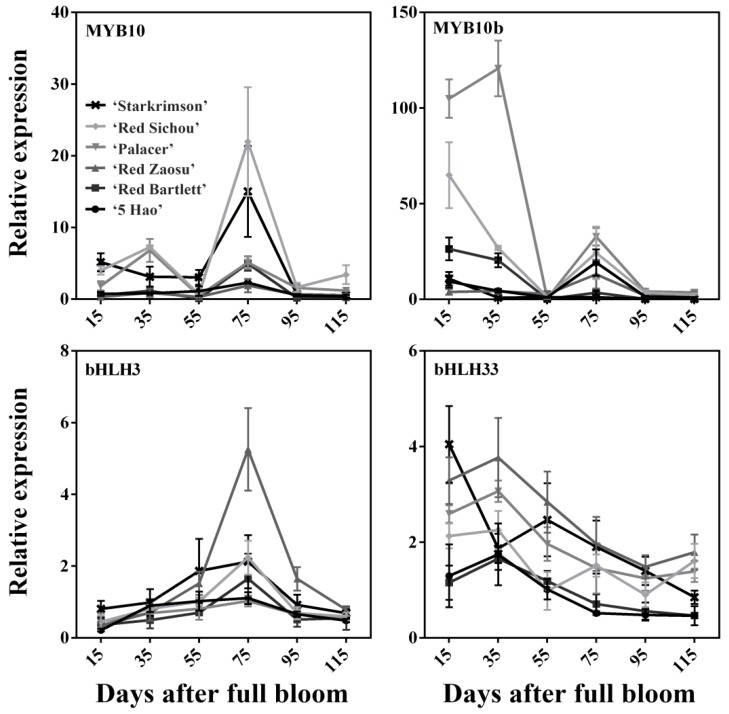
Relative expression levels of the anthocyanin biosynthesis-related transcription factor genes of six red pear cultivars at different stages of fruit development.

Four anthocyanin synthetic transcription factors, *MYB10*, *MYB10b*, *bHLH3,* and *bHLH33*, were selected and their expression levels determined during the stages of fruit development (Figure 3). The highest expression of *MYB10* occurred at 75 DAFB in the six cultivars, and overall it showed a tendency to first increase and then decrease, which was consistent with the trend of anthocyanin accumulation. The expression levels of *MYB10* in Starkrimson and Red Sichou were higher than in the other cultivars. The expression level of *MYB10b* was higher at the early stages of fruit development in Red Sichou, Palacer, Starkrimson, and Red Bartlett. However, *MYB10b* peaked at 75 DAFB in 5 Hao and Red Zaosu. The highest expression of *bHLH3* also appeared at 75 DAFB in the six cultivars, showing a tendency to first increase and then gradually decrease, like the trend of the anthocyanin accumulation. *bHLH33* showed a tendency to slowly decrease throughout the fruit’s developmental stages, contrary to the trend of the anthocyanin accumulation.

### 2.4. Expression Levels of Anthocyanins Synthesis-Related Transporter and Light-Responsive Genes

**Figure 4:**
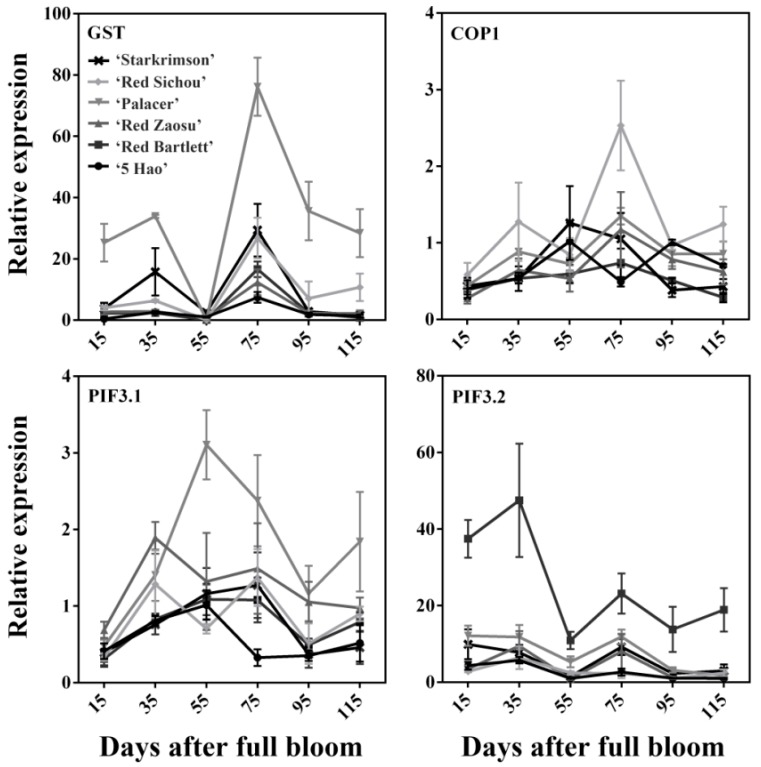
Relative expression levels of the anthocyanin biosynthesis-related transporter and light-responsive genes of six red pear cultivars at different stages of fruit development.

The expression levels of the transporter and light-responsive genes—*GST*, *COP1*, *PIF3.1*, and *PIF3.2*—involved in anthocyanin synthesis were also determined (Figure 4). The expression level of *GST* reached maximum values at 75 DAFB, having shown a tendency to increase first and then decrease. The expression level of *COP1* did not change significantly during the stages of fruit development. The expression level of *PIF3.1* showed a rise–drop trend and peaked when the highest anthocyanin content occurred at 35 DAFB in Red Zaosu. However, the expression level of *PIF3.1* showed a rise–drop–rise trend in 5 Hao, Red Bartlett, Starkrimson, and Palacer. The expression level of *PIF3.1* reached a peak at 55 DAFB in 5 Hao, Red Bartlett and Palacer, and reached a peak at 75 DAFB in Red Sichou and Starkrimson. *PIF3.2* had an initial peak in the early developmental stages in all cultivars, and had a second peak at 75 DAFB in ‘Red Zaosu’, Starkrimson, and Palacer. In addition, the expression of *PIF3.2* in Red Bartlett was much higher than in the other cultivars.

### 2.5. Correlation Analysis of Anthocyanin Contents and Gene Expression Levels 

In all structural genes, the anthocyanin content of 5 Hao was significantly positively correlated with the expression levels of *PAL*, *CHS*, *F3H*, *DFR1*, *DFR2*, and *UFGT2*, with *CHS*, *F3H*, and *UFGT2* being the three most relevant. The anthocyanin content of Red Bartlett was significantly positively correlated with the expression levels of *CHS*, *CHI*, *F3H*, *DFR1*, *ANS1*, *UFGT1*, and *UFGT2,* the highest of which was *F3H.* Red Zaosu had significant positive correlations with the expression levels of *PAL*, *CHS*, and *ANS2*, anthocyanin accumulation did not show significant correlation with *UFGT1* and *UFGT2*. The anthocyanin content of Palacer showed significant positive correlations with the expression levels of *CHS*, *CHI*, *F3H*, and *ANR,* but did not show significant positive correlations with *UFGT1* and *UFGT2*. The anthocyanin content of Red Sichou showed significant positive correlations with the expression levels of *PAL*, *CHS*, *CHI*, *F3H*, *ANR*, *DFR2,* and *UFGT2*, with *CHS*, *CHI*, and *F3H* being the three most relevant. In Starkrimson, on the other hand, there was a significant positive correlation between anthocyanin content and the expression of *F3H*, *DFR1*, *UFGT1* and *UFGT2*, and the most relevant genes were *UFGT1* and *UFGT2* (Table 1).

The expression level of *MYB10* was significantly positively correlated with the anthocyanin contents of 5 Hao, Red Sichou, Palacer, Starkrimson, and Red Bartlett, while the expression level of *MYB10b* was only significantly positively correlated with the content of 5 Hao. The expression level of *bHLH3* was significantly positively correlated with the anthocyanin contents of all of the cultivars, except for Red Zaosu. The expression level of *bHLH33* was not significantly correlated with the anthocyanin contents of the six cultivars, however it showed a positive correlation to the content of Red Zaosu (Table 1).

The expression level of *GST* was significantly positively correlated with the anthocyanin contents of 5 Hao, Starkrimson, and Red Bartlett, and it also showed positive correlations with the contents of the other three cultivars. *COP1*’s expression level showed a significantly positive correlation with the anthocyanin contents of Palacer, Starkrimson, and Red Bartlett. *PIF3.1* showed significantly positive correlations with the contents of Red Zaosu, Red Sichou and Starkrimson, while that of *PIF3.2* only showed a positive correlation with the anthocyanin content of Red Zaosu (Table 1).

### 2.6. Classification and Analysis of Important Genes in the Six Red Pear Cultivars

**Figure 5:**
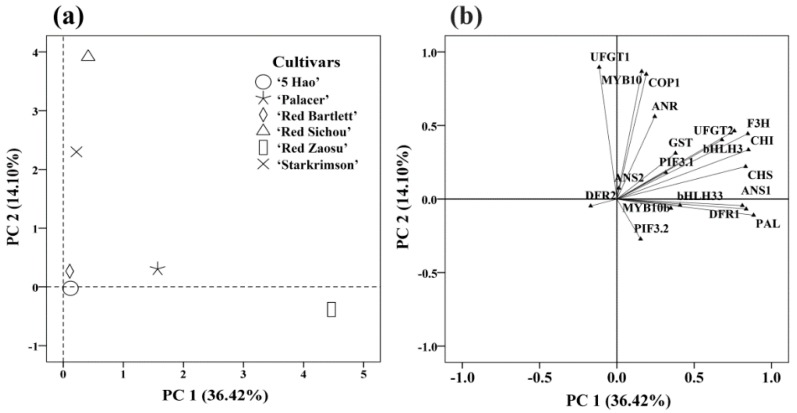
Principal component analysis of the skin of six red pears at different stages of fruit development. (**a**) Discrimination of six cultivars. (**b**) Loading plots of metabolites and transcripts for the first two principal components (PC1 and PC2).

To identify different coloring patterns and the responsible genes in six red pear cultivars, a Principal component analysis (PCA) was conducted on the anthocyanin accumulation (Figure 5a). Because the anthocyanin content peaked at 75 DAFB, we selected the quantitative data of genes at this developmental stage for analysis. The PCA readily discriminated the six cultivars using the first two principal components (PCs), which explained 50.52% of the total variance. PC1 discriminated Red Zaosu, mainly through differences in the expression levels of *PAL*, *CHI*, *F3H*, *ANS1*, *CHS*, *DFR1*, *UFGT2*, *bHLH33*, *bHLH3*, *GST*, *MYB10b*, and *PIF3.1*. PC2 discriminated Red Sichou and Starkrimson, based mainly on the transcriptional levels of *CHI*, *F3H*, *UFGT2*, *bHLH3*, *GST*, *UFGT1*, *MYB10*, *COP1*, and *ANR* (Figure 5b; Table S3). Palacer, 5 Hao, and Red Bartlett were discriminated by the two PCs. *CHI*, *F3H*, *UFGT2*, *bHLH3*, and *GST* were determined by the two PCs, indicating that these genes had commonalities in the six cultivars and could not be distinguished. The genes in PC1, *PAL*, *ANS1*, *CHS*, *DFR1*, *bHLH33*, *MYB10b*, and *PIF3.1*, were particular to Red Zaosu, indicating that their expression levels resulted in the unique anthocyanin accumulation pattern of Red Zaosu compared with the patterns of the other cultivars. The genes in PC2, *UFGT1*, *MYB10*, *COP1*, and *ANR*, were particular to the anthocyanin accumulation patterns of Red Sichou and Starkrimson.

**Figure 6:**
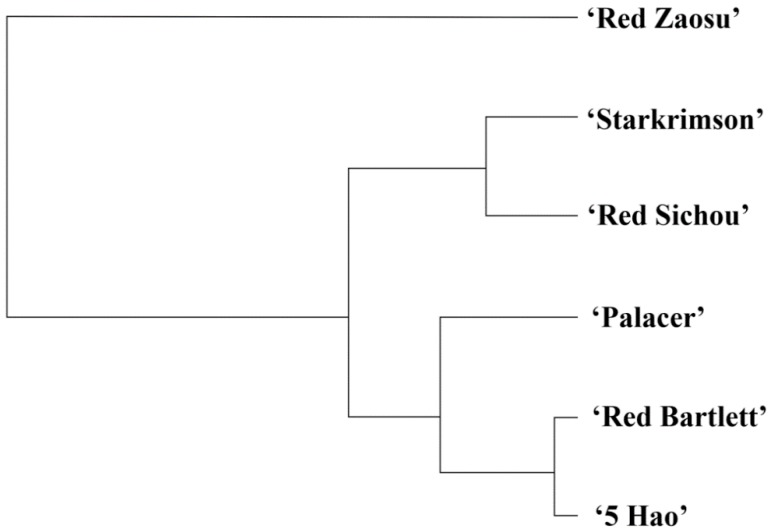
Dendrogram of the six red pear cultivars derived from the hierarchical cluster analysis.

To further classify the six cultivars, we performed a hierarchical cluster analysis according to the trends of anthocyanin accumulation (Figure 6). The six cultivars were divided into two groups. The European pears (Palacer, Red Bartlett, Starkrimson and Red Sichou) and the interspecific hybrid (5 Hao) were clustered into one group. Palacer, 5 Hao, and Red Bartlett were grouped on one branch, and the relationship between 5 Hao and Red Bartlett was the closest. Starkrimson and Red Sichou were grouped on to another branch. In addition, Red Zaosu (Asian pear) was clustered into one group, which had a special position in the dendrogram. The clustering results were basically consistent with the PCA (Figure 6).

## 3. Discussion

*UFGT* and *ANS* are downstream genes of anthocyanin biosynthesis [28]. Litchi, grape, apple, and other studies have confirmed the positive correlation between *UFGT* and anthocyanin accumulation [36,37,38]. In pears, the *UFGT* may be the decisive gene for the anthocyanin synthesis pathways [39,40,41]. There are also other studies showing that *UFGT* activity is not significantly correlated with anthocyanin accumulation. In the early development of pear fruits, anthocyanin contents and *UFGT* activity increased, while *UFGT* activity did not decrease when anthocyanin content decreased near maturity [31]. *PbUFGT1* may be the key factor because its transcriptional level increased rapidly after picking in Red Zaosu, while *PbUFGT2* showed a quite low transcriptional level after picking [42]. Moreover, the anthocyanin accumulation and transcriptional level of *UFGT2* are highly correlated [43]. In our study, *UFGT1* was only significantly positively correlated with the anthocyanin content of Red Bartlett and Starkrimson. However, *UFGT2* was significantly positively correlated with the anthocyanin contents of multiple cultivars (5 Hao, Red Bartlett, Red Sichou, and Starkrimson) and positively correlated with those of Palacer and Red Zaosu (Figure 2; Table 1). Therefore, we speculate that *UFGT2* may be an important gene for anthocyanin synthesis in the six pear cultivars. In red pears, *ANS* may also be a key gene for the anthocyanin synthesis pathways [39,40]. The expression trend of *ANS1* and *ANS2* was inconsistent with the trend of anthocyanin accumulation. In addition, the expression levels of *ANS1* and *ANS2* showed negative correlations with the anthocyanin contents in 5 Hao and Palacer (Figure 2; Table 1). PCA also showed that *ANS1* and *ANS2* were not key genes for anthocyanin accumulation in 5 Hao and Palacer (Figure 5). Analysis of the structural genes of anthocyanin in the kiwifruit Hongyang--*F3H2* and *F3GT1*—shows that they may be important for the formation of red flesh during fruit development [44]. In apple skin, the expression level of *F3H* is consistent with anthocyanin accumulation, showing a positive correlation [37]. Our results also showed that *F3H* is positively correlated with anthocyanin accumulation.

*MYB10* plays an important role in the regulation of anthocyanins in Rosaceae [45,46]. In pears, *PyMYB10* and *PbMYB10b* play important roles in regulating anthocyanin biosynthesis [32,33,34]. Here, the expression level of *MYB10* had a significant positive correlation with the anthocyanin contents of the cultivars, except Red Zaosu. *MYB10b* only had a significantly positive correlation with the anthocyanin content of 5 Hao (Table 1). Thus, *MYB10* may be a key transcription factor involved in anthocyanin synthesis. In addition, the overexpression of *MYB10/bHLH3* increases the production of anthocyanins in peaches by up-regulating *CHS*, *DFR*, and *UFGT* [47]. In apples, the interactions of *MdbHLH3*, *MdbHLH33*, and *MdMYB10* induce anthocyanin accumulation, and the binding of *MdMYB10/MdbHLH3* activates the *DFR* promoter much more than the binding of *MdbHLH33* does [48]. Our study showed the same result. The expression level of *bHLH33* did not show significant positive correlations with the anthocyanin levels in any of the cultivars, and only showed positive correlations to the levels in Red Zaosu, Palacer, and Red Sichou (Table 1). However, *bHLH3* showed positive correlations with the anthocyanin accumulations in all of the cultivars. Thus, *bHLH3* may play an important role in regulating anthocyanin synthesis in red pears. *MYB10* and *bHLH3* are crucial transcription factors involved in the anthocyanin biosynthesis of red pears.

Our study showed that some genes exhibited lower expression level at 55 DAFB. It is speculated that the rate of anthocyanin synthesis was affected by the high temperature or physiological reasons. High temperatures (greater than 30 °C) inhibit the expression of anthocyanin activators and related structural genes (*CHS*, *F3H*, *DFR*, *LDOX* and *UFGT*) in grapes, which affects anthocyanin synthesis [49].

*GST* has a positive correlation with anthocyanin accumulations in different species [24,25,26,27]. Our study corroborated these results. *GST* played an important role in the accumulation of anthocyanins and was positively correlated with the anthocyanin contents of the other three cultivars (Table 1). Many light-responsive genes are involved in anthocyanin synthesis. In apples and arabidopsis, COP1 degrade the MYB protein, which inhibits fruit coloration under dark conditions [20,50,51]. In this study, *COP1* did not show a negative correlation with the anthocyanin contents of the cultivars, except for 5 Hao (Table 1). This result differs from those of previous reports, which might be the result of the different regulatory mechanisms of anthocyanin biosynthesis in different species. Overexpression of *PIF3* can promote anthocyanins accumulation and can activate promoters of structural genes in the anthocyanin synthetic pathway [22]. *PIF3.1*’s expression level was positively correlated with the anthocyanin contents of the cultivars, except for 5 Hao (Table 1). This may be because 5 Hao is a hybrid of *P. bretschneideri* × *P. communis*. The expression level of *PIF3.2* was only significantly positively correlated with the anthocyanin contents of Red Zaosu. This indicates that *PIF3.1* plays an important role in the anthocyanin biosynthesis of red pears.

The PCA divided the six red pear cultivars into three types according to the dynamic expression levels of anthocyanin synthesis-related genes. Red Zaosu was separated into PC1 (Figure 5). The hierarchical cluster analysis also separated Red Zaosu into one group, showing that it has a distant relationship with the other five cultivars (Figure 6). There was a significant difference between the phenotype of Red Zaosu, in which the skin shows red-green stripes, and those of the other cultivars (Figure 1). Red Zaosu is the red bud mutation of Zaosu. Zaosu is a hybrid offspring of Mishirazi and Pingguoli (*P. pyrifolia* × *P. ussuriensis*). Mishirazi is native to Japan, with unknown parents, and is considered to be a hybrid offspring of *P. communis × P. pyrifolia* [52]. The PCA and cluster analysis separated 5 Hao and Red Bartlett into one group (Figure 5 and Figure 6). Many of their genes (*CHS*, *F3H*, *MYB10*, and *bHLH3*) had the same expression trends, and they all showed significant positive correlations with the anthocyanin contents (Table 1). Red Bartlett is the red bud mutation of Bartlett (*P. communis*), and 5 Hao is the hybrid offspring of Bartlett and an Asian pear. Their anthocyanin contents first increased and then decreased, with maximum accumulations at 75 DAFB. At that time, their anthocyanin contents dropped sharply as they entered the late stages of fruit development, and the fruits’ color faded. 

## 4. Materials and Methods

### 4.1. Plant Materials and Experimental Treatments

The sample collection list is shown in Table S1. The six cultivars were collected from an orchard in Meixian, Shaanxi Province, China, in 2017. The pears were collected at 15, 35, 55, 75, and 95 days after full bloom (DAFB) (Table S1). Fifteen fruits acted as biological repeats, with three biological replicates per period. For each sample, the peels of the fruits were removed, immediately frozen in liquid nitrogen and stored at −80 °C for further use. 

### 4.2. Anthocyanin Analysis

The total anthocyanin extraction was carried out as described by Giusti and Wrolstad [53], with slight modifications. Samples (0.2 g) were rapidly ground into a powder in liquid nitrogen, and then 1% HCL–methanol solution (5 mL) was added. Specific extraction steps and the method of calculation of the anthocyanin content are in accordance with the method of Wang et al. [24]. The anthocyanin content was determined spectrophotometrically. The absorbance of each extract was measured at 520 nm and 700 nm with a UV-Visible spectrophotometer (UV-1700, Kyoto, Japan). The total anthocyanin content was expressed as mg/kg fresh weight (FW). The value used for each sample was the mean of three independent biological replicates.

### 4.3. Quantification of Genes Expression 

Total RNA from the ground sample was isolated according to Reid et al. [54]. RNA isolation was followed by a DNase I treatment (Tiangen, Beijing, China). The RNA concentration and quality were detected by UV spectrophotometry and by running samples on a 1.2% agar ethidium bromide-stained gel. Then, 1 μg of total RNA was reverse-transcribed to cDNA using a PrimeScript RT reagent kit with gDNA Eraser (TaKaRa, Dalian, China). Every quantitative real-time PCR (qRT-PCR) was performed in three replicates on an Icycler iQ5 (Bio-Rad, Berkeley, CA, USA) with the SYBR Premix Ex Taq II (TaKaRa, Dalian, China) according to the manufacturer’s instructions. Reaction mixes (20 μL) included 10 μL of SYBR Green Supermix (TaKaRa, Dalian, China), 2 μL of diluted cDNA, and 0.4 μM of each primer. The specific annealing of the oligonucleotides was verified by dissociation kinetics at the end of each PCR run. The efficiency of each primer pair was quantified using a PCR-product serial dilution. All biological samples were assayed in technical triplicates. The EF1 and GAPDH genes were used as internal standards and for normalizing the expression. Data were analyzed using iQ5 2.0 software (Bio-Rad) with the 2^−ΔΔCT^ algorithm. All primer sequences are listed in Table S2.

### 4.4. Statistical Analyses

Statistical analyses were performed using Microsoft Excel 2010 and GraphPad Prism 5.01. (GraphPad Prism Software, San Diego, CA, USA). Each value is the mean ± standard deviation of three independent biological replicates.

Data were analyzed with multivariate analysis methods using SPSS 23.0 software (SPSS, Chicago, IL, USA). The Pearson correlation analysis (r) of the anthocyanin content and gene transcript levels was used to test for statistical significance (P). Statistically significant correlations were based on *p* ≤ 0.05. A PCA was conducted on the mean-centered and scaled data to investigate the discrimination of different cultivars using gene transcript levels. The ability of each principal component (PC) to explain the corresponding variables (select data with correlation coefficients greater than the absolute value 0.3) is shown in Supplementary Table S3. A dendrogram for the six pear cultivars was derived from the results of the real-time PCR by a hierarchical cluster analysis.

## 5. Conclusions

The peak accumulations of anthocyanin in all of the cultivars occurred during the middle stages of fruit development. The different expression levels of the structural genes *F3H* and *UFGT2* were important for anthocyanin accumulation in the six cultivars. The transcription factors *MYB10* and *bHLH3* were also important for anthocyanin accumulation. A PCA and hierarchical cluster analysis were used to distinguish the types of cultivars and the genes important for each type of anthocyanin accumulation pattern. The anthocyanin contents of all six cultivars showed a rise–drop tendency. The six cultivars were divided into three groups, with Red Zaosu (Asian pear) clustered into one group, Red Sichou and Starkrimson (European pears) clustered into another group, and the Palacer, Red Bartlett (European pears), and 5 Hao (interspecific *Pyrus* hybrid) clustered into a third group. The potential mechanisms behind the regulation of anthocyanin synthesis are complex, and further studies of related gene functions still need to be explored.

## Figures and Tables

**Table 1:** Correlation analysis of the anthocyanin contents of six red pear cultivars and anthocyanin synthesis-related gene expression levels.

Anthocyanin	5 Hao	Red Bartlett	Red Zaosu	Palacer	Red Sichou	Starkrimson
*PAL*	0.590 *	0.09	0.534 *	0.178	0.571 *	0.058
*CHS*	0.962 **	0.779 **	0.639 *	0.611 *	0.861 **	−0.102
*CHI*	0.382	0.759 **	0.473	0.692 **	0.830 **	0.416
*F3H*	0.943 **	0.882 **	0.504	0.556 *	0.850 **	0.557 *
*DFR1*	0.794 **	0.759 **	0.499	−0.003	0.358	0.586 *
*DFR2*	0.578 *	0.451	0.005	0.033	0.648 **	−0.137
*ANS1*	−0.278	0.578 *	0.429	−0.47	0.492	0.458
*ANS2*	−0.221	0.29	0.553 *	−0.411	0.336	0.257
*ANR*	−0.28	0.227	0.065	0.590 *	0.523 *	−0.049
*UFGT1*	0.301	0.792 **	−0.073	0.231	0.449	0.754 **
*UFGT2*	0.949 **	0.725 **	0.513	0.459	0.775 **	0.767 **
*MYB10b*	0.809 **	−0.169	0.358	0.017	−0.065	−0.222
*MYB10*	0.953 **	0.875 **	0.392	0.745 **	0.679 **	0.725 **
*bHLH3*	0.744 **	0.747 **	0.311	0.639 *	0.680 **	0.751 **
*bHLH33*	−0.273	−0.055	0.47	0.023	0.219	−0.009
*GST*	0.948 **	0.908 **	0.399	0.477	0.513	0.773 **
*COP1*	−0.299	0.860 **	0.071	0.670 **	0.278	0.716 **
*PIF3.1*	−0.294	0.488	0.684 **	0.379	0.791 **	0.692 **
*PIF3.2*	−0.046	−0.04	0.783 **	0.143	0.481	0.297

All data are means based on three replicates. Levels of significance: * *p* ≤ 0.05; ** *p* ≤ 0.01.

## Supplementary Materials

The following are available online at https://www.mdpi.com/2223-7747/8/4/100/s1, Table S1: Sample site of six red pear cultivars, Table S2: Sequences of primers used for qRT-PCR analysis, Table S3: Rotated Component Matrix, Figure S1: A schematic presentation of the flavonoid biosynthetic pathway leading to anthocyanins.
