# Supplementary material for: Differences among the Anthocyanin Accumulation Patterns and Related Gene Expression Levels in Red Pears
Source: Plants (Basel). 2019 Apr 16;8(4):100. doi: 10.3390/plants8040100 (PMC6524040; doi:10.3390/plants8040100)
Supplement: Supplementary file 1 [file plants-08-00100-s001.pdf]

Supplementary Material

Table S1. Sample site of six red pear cultivars

No.	Cultivars	Species	Stages
a	'5 Hao'	Inter specific *Pyrus* hybrid	15/35/55/75/95/115 DAFB
b	'Red Bartlett'	*Pyrus communis*	15/35/55/75/95/115 DAFB
c	'Red Zaosu'	*Pyrus. bretschneideri*	15/35/55/75/95/115 DAFB
d	'Palacer'	*Pyrus communis*	15/35/55/75/95/115 DAFB
e	'Red Sichou'	*Pyrus communis*	15/35/55/75/95/115 DAFB
f	'Starkrimson'	*Pyrus communis*	15/35/55/75/95/115 DAFB

Table S2. Sequences of primers used for qRT-PCR analysis

Gene/Copy	Gene ID.	Encoded protein		sequence of forward (5'-3') and reverse ®primers (5'-3')
PAL	GU390550.1	phenylalanine ammonia lyase	F	5'-TGTTCCAAGCTATCTGCCAGGGAA-3'
			R	5'-TGGAATGGAATGCAGCACTCAAGC-3'
CHI	NW_008988454.1	chalcone isomerase	F	5'-ATCGAGGGCAAGTTGGTGAA-3'
			R	5'-CCTGTGACGACGTCCTTGAT-3'
CHS	KF387513.1	chalcone synthase	F	5'-ACTGTCGAGGAAGTTCGCAA-3'
			R	5'-CGGGATATGTGGCTTGATCC-3'
F3H	NW_008988143.1	flavanone 3-hydroxylase	F	5'-TGGGTTCTGGAATGTGGCTATGGA-3'
			R	5'-TGGGAAGACATGGATCACCGTTCA-3'
DFR1	NW_008988041.1	Dihydroflavonol4-reductase	F	5'-GATTTCATCACCATTATCCCAACTC-3'
			R	5'-TGCCAACTTTGACATCAACGAG-3'
DFR2	NW_008988081.1	Dihydroflavonol4-reductase	F	5'-GCCCAAAGTTGTCCAATGTCT-3'
			R	5'-GCTTGTAAGTGAATCCCAAATCTG-3'
ANS1	MF489223.1	anthocyanidin synthase	F	5'-AGCTTCAACACCAAGTGCAAGCTC-3'
			R	5'-TTGGATGAAGGGAGGCTGGAGAAA-3'
ANS2	XM_009370901	leucoanthocyanidin dioxygenase	F	5'-AGCGACATACCAATTGAGCC-3'
			R	5'-CCTCGAGGGCCATTCAATCTT-3'
ANR	DQ251189.1	anthocyanidin reductase	F	5'-CATGTGGAGGATGTCTGCC-3'
			R	5'-GGGGGTATCTTTTGTTGAGG-3'
UFGT1	NW_008988899.1	anthocyanidin3-O-glucosyltransferase	F	5'-CTGGAACCTGAAGTTGTGAATCTG -3'
			R	5'-AGCCACTCTAAGCAACCACTATC-3'
UFGT2	NW_008988121.1	anthocyanidin 3-O-glucosyltransferase	F	5'-GGACACTATCGGAACTCAAGG-3'
			R	5'-AGGTCGAGTTCTTCGAAACAG-3'
MYB10	NW_008988216.1	R3R3 myb-type transcipt factor	F	5'-TCATCGACAAAGAATGGAA-3'
			R	5'-CTAACTGAAGGCTGGGACA-3'
MYB10b	NW_008988130.1	R3R3 myb-type transcipt factor	F	5'-AGATTTCACAGAGGACGAA-3'
			R	5'-CCCCTAACCCTAAACTAGA-3'
bHLH3	XM_018647540.1	basic helix-loop-helix protein	F	5'-ACTTCGGCAGTGGCGTTC-3'
			R	5'-TCACATTCTCGCGTTCAGCAA-3'
bHLH33	XM_009341833.2	basic helix-loop-helix protein	F	5'-GTGATGCAAACACTTAGGGAGC -3'
			R	5'-TTGCCACTCACGTTATCCTTCA-3'
GST	XM_009372312.2	glutathione S-transferase	F	5'-AAGTATGCAGGCCGTGGTC-3'
			R	5'-CAGGTCGTTGAAGTTGTGGC-3'
COP1	NW_008988230.1	E3 ubiquitin-protein ligase	F	5'- TTGCATGTTTCTCTCCGCCT-3'
			R	5'- GCCAGACATTGCAACAGGTG-3'
PIF3.1	XM_009367742.2	phytochrome interacting factor 3	F	5'-TGCAAGGAGCACATTTCCCT-3'
			R	5'-GGGAGCTAAGGGCGCATATT-3'
PIF3.2	XM_018649444.1	phytochrome interacting factor 3	F	5'-ATCCCAAGCGTCGTTTCCAT-3'
			R	5'-GTTACTGGATCTGGCCTCGG-3'
ACTIN			F	5'-CCATCCAGGCTGTTCTCTC-3'
			R	5'-GCAAGGTCCAGACGAAGG-3'

Table S3. Rotated Component Matrix[a]

	Component	
	**1**	**2**
PAL	0.885	−0.11
CHI	0.851	0.335
F3H	0.847	0.444
ANS1	0.837	−0.068
CHS	0.833	0.221
DFR1	0.812	−0.044
UFGT2	0.76	0.463
bHLH3	0.68	0.403
bHLH33	0.409	−0.042
GST	0.38	0.311
MYB10b	0.349	−0.062
PIF3.1	0.317	0.181
DFR2	−0.169	−0.047
UFGT1	−0.114	0.896
MYB10	0.16	0.867
COP1	0.19	0.849
ANR	0.245	0.561
PIF3.2	0.154	−0.272
ANS2	0.013	0.074

Extraction Method: Principal Component Analysis. Rotation Method: Varimax with Kaiser Normalization. [a] Rotation converged in 3 iterations.

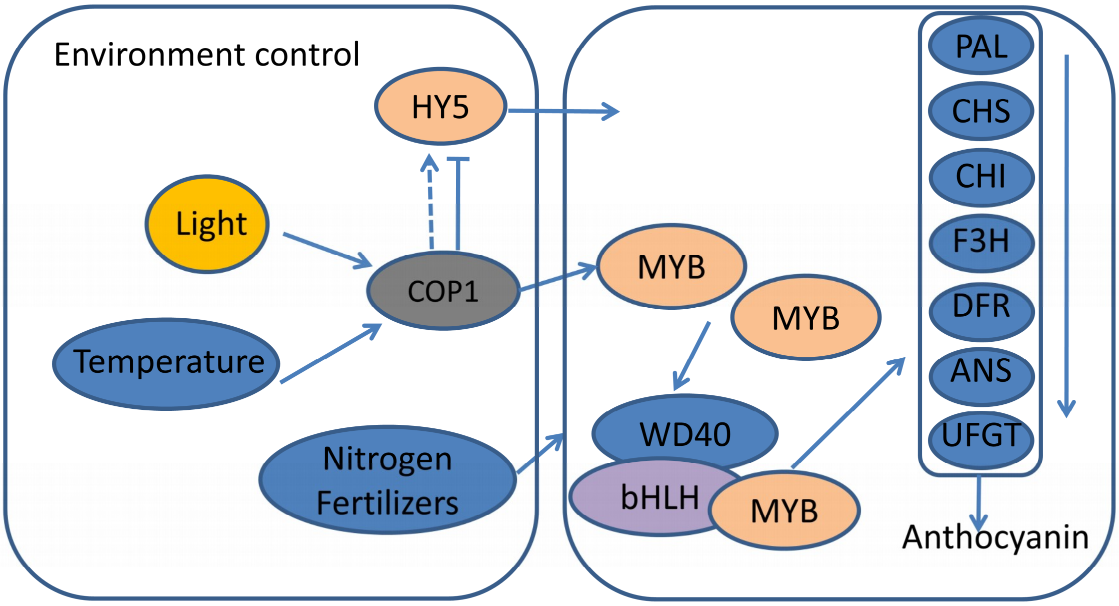

Figure S1. A schematic presentation of the flavonoid biosynthetic pathway leading to anthocyanins.
